# Individual-based simulation model for COVID-19 transmission in Daegu, Korea

**DOI:** 10.4178/epih.e2020042

**Published:** 2020-06-15

**Authors:** Woo-Sik Son, RISEWIDs Team

**Affiliations:** 1 National Institute for Mathematical Sciences, Daejeon, Korea

**Keywords:** COVID-19, Mathematical model, Model prediction, Infections, Korea

## Abstract

**OBJECTIVES:**

The aims of this study were to obtain insights into the current coronavirus disease 2019 (COVID-19) epidemic in the city of Daegu, which accounted for 6,482 of the 9,241 confirmed cases in Korea as of March 26, 2020, to predict the future spread, and to analyze the impact of school opening.

**METHODS:**

Using an individual-based model, we simulated the spread of COVID-19 in Daegu. An individual can be infected through close contact with infected people in a household, at work/school, and at religious and social gatherings. We created a synthetic population from census sample data. Then, 9,000 people were randomly selected from the entire population of Daegu and set as members of the Shincheonji Church. We did not take into account population movements to and from other regions in Korea.

**RESULTS:**

Using the individual-based model, the cumulative confirmed cases in Daegu through March 26, 2020, were reproduced, and it was confirmed that the hotspot, i.e., the Shincheonji Church had a different probability of infection than non-hotspot, i.e., the Daegu community. For 3 scenarios (I: school closing, II: school opening after April 6, III: school opening after April 6 and the mean period from symptom onset to hospitalization increasing to 4.3 days), we predicted future changes in the pattern of COVID-19 spread in Daegu.

**CONCLUSIONS:**

Compared to scenario I, it was found that in scenario III, the cumulative number of patients would increase by 107 and the date of occurrence of the last patient would be delayed by 92 days.

## INTRODUCTION

Coronavirus disease 2019 (COVID-19) is an infectious disease caused by the new coronavirus (2019-nCoV) [1]. It began spreading in Wuhan, China, in December 2019, and on January 14, 2020, the World Health Organization officially stated that there was a potential for inter-personal infection of COVID-19 in a limited range between families, and on January 22, 2020, the officials in Wuhan, China, reported that there was evidence of COVID-19 infection among people [2]. In Korea, the first patient was confirmed with COVID-19 on January 20, 2020. Thereafter, 30 confirmed cases appeared by February 16, 2020, and the rate of increase in confirmed cases was limited to one or two cases per day. However, the situation rapidly changed after the #31 confirmed case on February 18, 2020 in Daegu city. As of March 26, 2020, of the 9,241 confirmed cases in Korea, 6,482 confirmed cases occurred in Daegu, accounting for 70.1% of the total cases. Among them, 4,391 (67.7%) confirmed cases in Daegu were related to Shincheonji. Assuming there are 2.5 million citizens in Daegu, and 9,000 members are part of Daegu Shincheonji, the 2019-nCoV infection rate of Daegu citizens was 0.08%, while the infection rate of Daegu Shincheonji members was 48.78%, which is 583 times higher. This shows that the COVID-19 epidemic in Daegu was because the Shincheonji Church in Daegu became a hotspot, leading to the spread of COVID-19 in the Daegu community.

A compartmental model [3], which is widely used as a mathematical study model for the spread of infectious diseases, generally divides the entire population into several groups depending on the state of infection, but it is not an appropriate model to reproduce the hotspot and non-hotspot (communities that are not members of Shincheonji) groups that appear in Daegu. In addition, although a two-patch model [4], that divides the entire population into two groups, was proposed to include hotspots and non-hotspots in the compartmental model, there are difficulties in reproducing infections caused by close contact between the two groups.

To overcome the above problems, we simulated the COVID-19 spread in Daegu using an individual-based model suggested by Ferguson et al. [5]. In the individual-based model, each individual can be infected through contact with infected persons in households, workplaces/schools, and communities (religious and social gatherings), and for this, a virtual population of the same size as the population in Daegu was created. The purpose of this study was to use the individual-based model to understand the current COVID-19 epidemic in Daegu, predict the future spread, and analyze how the reopening of schools scheduled for April 6, 2020 will affect the spread of COVID-19.

## MATERIALS AND METHODS

**Figure 1.**
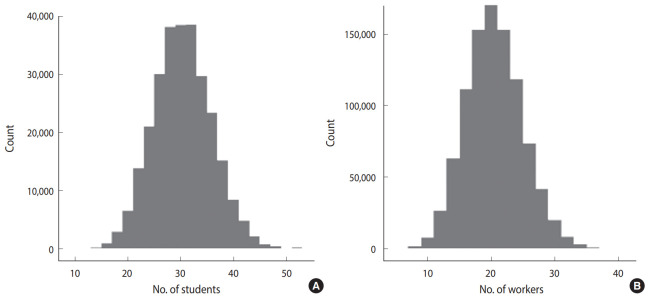
Histogram of the number of (A) students in the classroom and (B) workers in the office.

The individual-based model [5] simulates the spread of infectious diseases through close contact between people in households, workplaces/schools, and communities. Through the individual-based model, a virtual population group of the size of Daegu was created to understand the spread of COVID-19 in Daegu and predict future spread. Each individual in the virtual population group has information regarding the household, work/school, and community. We generated a virtual population of 2,171,000 people from the 2015 census 2% sample data of the MicroData Integrated Service (as of 2015) [6]. Household information and the status of students and workers were set using the age and commuting status of each individual listed in the census data. Assuming that close contact at work or school occurs in the same office or classroom, a student’s classroom ID and employee’s office ID were virtually generated as follows: the classroom ID was set by randomly selecting students of the same age, city, and district so that an average of 30 students were assigned to the same classroom. Figure 1A shows the histogram according to the number of students in each classroom in Daegu. The office ID was set by randomly selecting the workers in Daegu without the distinction of municipalities and assigning an average of 20 people to the same office. Figure 1B shows the histogram according to the number of workers in each office in Daegu.

**Figure 2.**
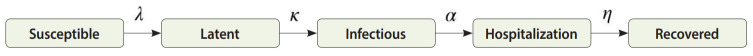
Compartmental structure of our epidemic model for coronavirus disease 2019 (COVID-19). The infection status is as follows (susceptible (S), latent (L), infectious (I), hospitalization (H), and recovered or dead (R)). Here, *λ* is the infection probability of the susceptible and *κ*, *α*, *η* are the latent period, period between symptom onset to confirmation, period from being confirmed to recovery, respectively.

Table 1 shows some of the virtual population groups created in this way. Each row represents one individual, and each column represents the attributes of the individual used to simulate the spread of the infectious disease. Individuals with the same household, classroom, and office IDs belonged to the same household, classroom, and office, respectively. If the classroom ID and the office ID are marked as NA, it means that the individual is neither a student nor worker. Items on hotspot indicates whether the individual is a member of Shincheonji or not, and 9,000 people were randomly selected from the population of Daegu and assigned to the hotspot. The infection status in the last column of Table 1 represents the infection status of each individual on a certain date, and the possible infection status were as follows: those susceptible to COVID-19 - susceptible (S); those in the latent stage after infection - latent (L); those that can infect the susceptible -infectious (I); those isolated after being confirmed with COVID-19 - hospitalization (H); and those that recovered or died from COVID-19 -recovered (R). Figure 2 is a diagram showing the change in each infection status. *λ* is the infection probability of the susceptible and was calculated as follows [5].

$$\lambda \;=\;\frac{\beta_{h}/3\cdot N_{h}^{I}}{{\left(N_{h}\right)}^{0.8}}\;+\;\frac{\beta_{w}/3\cdot N_{w}^{I}}{N_{w}}\;+\;\frac{\beta_{s}/3\cdot N_{s}^{I}}{N_{s}}\;+\;\frac{\beta_{hotspot}/3\cdot N_{hotspot}^{I}}{N_{hotspot}}$$

*β_h_* (*β_w_*, *β_s_*, *β_hotspot_*) is the probability of encountering an infected individual in the household (work, school, hotspot) and getting infected. We set this as *β_h_*=*β_w_*,*β_s_*=2*β_h_*, same as that set by Ferguson et al. [5]. *N_h_* (*N_w_*,*N_s_*, *N_hotspot_*) represents the total number of households (work, school, hotspot), and $N_{h}^{I}\;(N_{w}^{I},\;N_{s}^{I},\;N_{hotspot}^{I})$ represents the total number of those infected in households (work, school, hotspot). 1/*κ*=5.2 [7], 1/*α*=4.3 [8], and 1/*η*=14 are the average latent period (days), average period between symptom onset to confirmation (days), and average period from being confirmed with COVID-19 to recovery (days), respectively.

In February 1, 2020, 10 infected individuals in the hotspot were set as the initial confirmed patients. The #31 patient was confirmed with COVID-19 on February 18, 2020, but it was assumed that the symptoms started on February 7, 2020, and considering the latent period of COVID-19, the #31 patient was assumed to be infected from the initial patients of the hotspot on February 1st. The individual-based model was simulated on a daily basis, and it was assumed that the population inflow into Daegu using public transportation was minimal after the COVID-19 epidemic in Daegu. That is, it was assumed that there was no influx of new COVID-19 infections from other regions in Korea and from abroad.

### Ethics statement

This research is based on data which is open to public. Neither ethical approval of an institutional review board nor written informed consent we required.

## RESULTS

**Figure 3.**
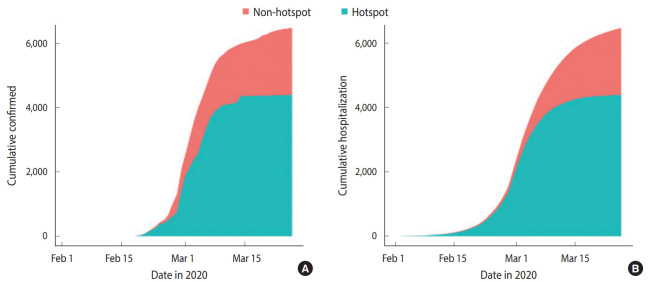
(A) Cumulative confirmed coronavirus disease 2019 (COVID-19) cases in the city of Daegu and (B) cumulative number of hospitalizations in the simulation. Here, we show the median of 100 simulation results for different random seeds.

The individual-based model and all parameter values except for *β_hotspot_* and *β_h_* are described in the Materials and Methods section. Data on the date of onset of symptoms in confirmed patients with COVID-19 are not currently available. We set *β_hotspot_* and *β_h_* to realize the confirmed COVID-19 patients in Daegu until March 26, 2020 (4,391 cases in the hotspot, 2,091 in the non-hotspot). In the individual-based model, changes in infection status are statistically simulated; that is, the changes in which a susceptible person becomes a latent person is realized as follows. Every day, a uniform random number between 0 and 1 is generated for all susceptible people, and if this value is less than lambda, which is the probability of becoming latent after infection, that susceptible person becomes a latent patient. Since the random number changes every time it is generated, the simulation results can be different even with the same parameters and initial patient settings. Therefore, it is necessary to check the distribution of simulation results using different random seeds rather than a single simulation. We performed 100 simulations using different random seeds and confirmed whether the median of this result reproduces the statistics of cumulative confirmed cases as of March 26, 2020. Among *β_hotspot_* and *β_h_*, the parameter with a higher determinant power for reproducing the cumulative confirmed cases in Daegu was *β_hotspot_*. When *β_hotspot_* was determined, the results of the cumulative confirmed cases were realized, and while adjusting for *β_h_*, a parameter value for reproducing the non-hotspot cumulative cases was found. The parameter results were *β_hotspot_*= 3.06 and *β_h_*= 0.33. This shows that the probability of infection between the hotspot and household and workplace differed by more than nine times. Figure 3 shows the cumulative confirmed cases and simulation results of the individual-based model by March 26, 2020. Since a list of members of Shincheonji Church and the screening and quarantining of them for COVID-19 infection began at the end of February, in the above simulation, *β_hotspot_*= 0 as of February 29, 2020. In addition, 1/*α* was set to 2.7 instead of 4.3 after February 29, 2020 to reflect the effect of the shortened 1/*α*, the average period from symptom onset to confirmation, after massive screening tests for the Shincheonji Church members.

The statistics of the confirmed cases as of March 26, 2020 were reproduced, and to predict the spread of COVID-19 in Daegu thereafter, we considered the three following scenarios.

- Scenario I: Maintaining vacations in elementary/middle/high schools

- Scenario II: Reopening of elementary/middle/high schools on April 6, 2020

- Scenario III: Reopening of elementary/middle/high schools on April 6, 2020 & after April 6, 2020, 1/*α*, the average period from symptom onset to confirmation, increases again to 4.3 days

**Figure 4.**
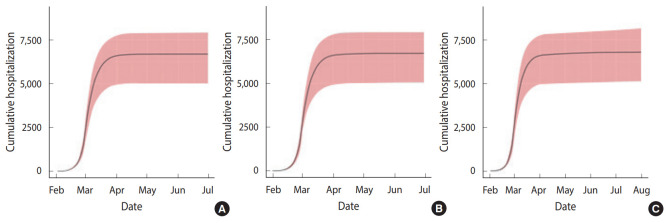
Cumulative number of hospitalization cases in the simulation. Here, we show the median and 5^th^ to 95^th^ percentile range for (A) scenario I, (B) scenario II, and (C) scenario III.

**Figure 5.**
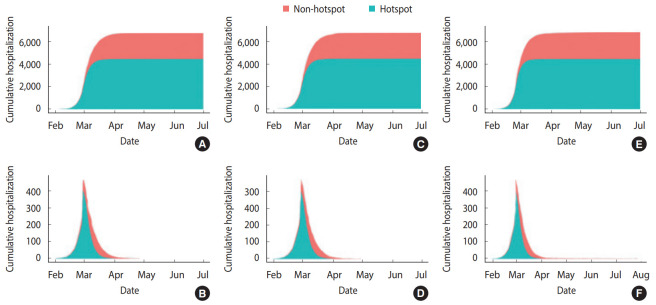
Cumulative and daily hospitalization cases in the simulation (A, B) scenario I, (C, D) scenario II, and (E, F) scenario III. Here, we show the median of 100 simulation results for different random seeds.

The reopening of elementary/middle/high schools on April 6, 2020, mentioned in scenarios II and III, is the current plan as of March 26, 2020. The assumption of scenario III that the average period from symptom onset to confirmation would increase again to 4.3 days considers the students’ relatively passive expression of symptoms after the reopening of schools. The individual-based model simulation results for the above three scenarios are shown in Table 2, Figures 4 and 5. In scenario I, the number of cumulative confirmed cases in Daegu was 6,677 (4,394 in hotspot, 2,322 in non-hotspot), and the last newly confirmed cases occurred on April 26, 2020. In scenario II, the number of cumulative confirmed cases in Daegu was 6,716 (4,394 in the hotspot, 2,322 in the non-hotspot) and compared with scenario I (based on the median), 39 Daegu citizens were additionally infected (non-hotspot, not a member of Shincheonji). The last newly confirmed cases occurred on May 3, 2020, 7 days later than in scenario I. In scenario III, the number of cumulative confirmed cases were 6,784 (4,394 in hotspot, 2,390 in the non-hotspot), and 107 additional citizens of Daegu that were not members of Shincheonji were infected. The last newly confirmed cases occurred on July 27, 2020, 92 days later than in scenario I. Figure 4 shows the cumulative daily confirmed cases for each scenario and the interval except for the median value and the top and bottom 5% of 100 simulations using different random seeds. Figure 5 shows the median values for cumulative confirmed cases in the hotspot and non-hotspot.

## DISCUSSION

The individual-based model was selected to explain the characteristics demonstrated by the spread of COVID-19 in Daegu, specifically, that the cumulative infection rate of Shincheonji members was about 583 times higher than that of non-Shincheonji members. Compared with the compartmental model which is widely used as a mathematical model for the spread of infectious diseases [3], the individual-based model simulates the transmission of infectious diseases through close contact among people using the socio-demographic information of each individual (household, workplace/school, community such as religious and social gatherings). Therefore, it has the advantage of being able to analyze the effect of quarantine policies, such as closing schools and implementing shifts at work, in preventing infection spread in more detail.

Using the individual-based model, we reproduced the cumulative COVID-19 confirmed cases of Daegu until March 26, 2020. The number of newly confirmed cases per day in Daegu sharply increased from February 21, 2020, after the #31 confirmed case, and the largest number of confirmed cases was reported on February 29, 2020, with 656 cases. Since then, and after March 11, 2020 with 131 confirmed cases, a decreasing trend was maintained with less than 100 confirmed cases per day. However, it cannot be said that the rapid increase in the number of confirmed cases during this period reflects the actual rate of COVID-19 spread in Daegu. Because of the intensive large-scale screening of the members of Shincheonji, it is likely that the rate increased more steeply than the actual rate of spread because more confirmed cases arose in a short period of time compared with the previous order and rate of infection. Since data on the date of symptom onset in patients in Daegu are not available, the results of this study using the cumulative data may be different from the actual COVID-19 transmission patterns in Daegu. If the data on the date of symptom onset are collected in the future, a follow-up study using this information should be conducted. In addition, because we did not assume any other additional group infections, such as in nursing homes, other than Shincheonji, this study may show a different pattern from the actual transmission of COVID-19.

The above results assume that newly infected COVID-19 cases in Daegu did not come from abroad or other regions in Korea. For a more accurate prediction and analysis of the effect of quarantine policies, such simulations should be expanded to reflect the whole country and consider the entry of latent patients from other regions and abroad. Studies on this are currently under way.

## Figures and Tables

**Table 1.** Each row of the synthetic population data represents one individual, and each column represents the attributes of the individual used to simulate the spread of coronavirus disease 2019 (COVID-19)^1^

Individual ID	House-hold ID	Age (yr)	Class-room ID	Office ID	Hotspot	Infectious status
1	1	48	NA	3	False	S
2	1	44	NA	NA	True	I
3	1	15	2	NA	False	S
4	2	45	NA	3	False	S
5	2	43	NA	NA	False	S
6	2	17	25	NA	False	S
7	3	51	NA	5	False	S
8	3	50	NA	NA	False	S
9	3	17	25	NA	False	S

NA, not available; S, susceptible; I, infectious.

1 Individuals with the same household, classroom, and office IDs belong to the same household, classroom, and office, respectively. If the classroom or office ID is NA, it means that she/he is not a student or worker. The hotspot indicates whether individuals are a member of Shincheonji. A detailed description of the infection status is shown in Figure 2.

**Table 2.** Cumulative number of hospitalization cases and hospitalization date of the last patient for each scenario in 2020

	Scenario I	Scenario II	Scenario III
	School closing	School opening after Apr 6	School opening after Apr 6 & the mean period from symptom onset to hospitalization increases to 4.3 d
Parameters	*β_s_* = 0, $\frac{1}{\alpha }\;=\;\{\begin{array}{c} 4.3\;(-\mathrm{Feb}\;28)\\ 2.7\;(\mathrm{Feb}\;29-) \end{array}\}\;$	*β_s_* = 2*β_h_*, $\frac{1}{\alpha }\;=\;\{\begin{array}{c} 4.3\;(-\mathrm{Feb}\;28)\\ 2.7\;(\mathrm{Feb}\;29-) \end{array}\}\;$	*β_s_* = 2*β_h_*, $\frac{1}{\alpha }\;=\;\{\begin{array}{c} 4.3\;(-\mathrm{Feb}\;28)\\ 2.7\;(\mathrm{Feb}\;29-\mathrm{Apr}\;5)\\ 4.3\;(\mathrm{Apr}\;6-) \end{array}\}\;$
Cumulative no. of hospitalization cases	6,677, (median)	6,716 (compared with scenario I + 39 cases)	6,784 (compared with scenario I + 107 cases)
Hospitalization date of the last patient	Apr 26	May 3 (compared with scenario I +7 d)	Jul 27 (compared with scenario I + 92 d)

## SUPPLEMENTARY MATERIALS

Korean version is available at http://www.e-epih.org/.
