# Supplementary material for: Individual-based simulation model for COVID-19 transmission in Daegu, Korea
Source: Epidemiol Health. 2020 Jun 15;42:e2020042. doi: 10.4178/epih.e2020042 (PMC7644934; doi:10.4178/epih.e2020042)
Supplement: Supplementary file 1 [file epih-42-e2020042-suppl.docx]

Title: Individual based simulation model for COVID-19 transmission in Daegu metropolitan city, South Korea

Type of Manuscript: Methods

Authors: Woo-Sik Son and RISEWIDs Team

*Full list of the members of Research and Development on Integrated Surveillance System for Early Warning of Infectious Diseases (RISEWIDs) team is provided in the acknowledgments.

Affiliations: National Institute for Mathematical Sciences, 34047 Daejeon, South Korea

This research was supported by Government-wide R&D Fund project for infectious disease research (GFID), Republic of Korea (grant number: HG18C0088).

Correspondence: Woo-Sik Son, National Institute for Mathematical Sciences, 34047 Daejeon, South Korea, wsson@nims.re.kr

Running title: Analysis of the effect of school closing

Conflicts of interest: The authors declare that they have no competing interests.

초록

목적: 2020년 3월 26일 현재, 국내 9,241명의 COVID-19 확진자 중 대구 지역 확진자가 6,482명이고, 이 중 신천지 관련 확진자는 4,391명이다. 대구 지역의 COVID-19 유행은 신천지 교인 집단이 hotspot이 되어 지역사회로 전파된 양상을 보인다. 지금까지의 대구 지역 COVID-19 유행을 이해하고, 향후 확산을 예측, 그리고 4월 6일로 예정된 개학이 어떤 영향을 미칠지 분석하고자 한다.

방법: Individual based model을 이용하여, 대구의 COVID-19 확산을 시뮬레이션 하였다. 각 개인은 가구, 직장/학교, 공동체 (종교 및 친목 모임)에서 감염자와 접촉을 통하여 감염될 수 있다. 인구센서스 샘플 데이터를 이용하여 대구 인구와 동일한 크기의 가상 인구 집단을 생성하였으며, 각 개인에게 가구, 직장/학교, 공동체 정보를 부여하였다. 대구 전체 인구 중 무작위로 9,000명을 선택하여 hotspot으로 설정하였다. 시뮬레이션은 일일 단위로 진행되었으며, 국내 다른 지역과의 인구 이동은 고려하지 않았다.

결과: Individual based model을 이용하여 2020년 3월 26일까지의 대구 COVID-19 누적 확진 결과를 재현하였으며, hotspot과 non-hotspot이 서로 다른 감염 확률을 갖고 있음을 확인하였다. 서로 다른 세 가지 시나리오 (I; 초/중/고 방학 유지, II; 4월 6일 초/중/고 개학, III; 초/중/고 개학 & 증상 발현 후 확진까지의 기간 증가)에 대해서 대구 지역 COVID-19 향후 확산이 어떻게 변화하는지 예측하였다. 시나리오 I의 경우, 대구 누적 환자는 6,677, 마지막 신규 환자 발생일은 4월 26일이다. 시나리오 II는 누적 환자 39명 증가, 마지막 신규 환자 발생일은 5월 3일이다. 시나리오 III는 누적 환자 107명 증가, 마지막 신규 환자 발생일은 7월 27일이다.

결론: 세 가지 시나리오에 대한 분석 결과, 4월 6일로 예정된 초/중/고 개학을 하고 증상 발현 후 확진까지의 기간이 4.3일로 복원될 시나리오 III의 경우, 방학을 계속 유지하는 시나리오 I의 경우와 비교하여 누적 환자 증가 107명, 신규 환자 발생일은 92일 지연되는 것을 확인할 수 있었다.

중심어 (Keywords)

COVID-19, Individual based model, Non-pharmaceutical intervention

서론 (introduction)

코로나바이러스감염증-19 (COVID-19)는 신종코로나바이러스 (SARS-CoV-2)에 의한 감염병이다 [1]. 2019년 12월 중국 우한 지역으로부터 확산이 시작되었으며, 2020년 1월 20일 중국 보건당국에 의하여 사람 사이의 감염이 처음으로 확인되었다 [2]. 국내에서는 2020년 1월 20일 COVID-19 첫 환자가 확진 되었다. 이후 2월 16일까지 30명의 확진자가 발생하였고, 확진자 증가 추세는 하루 한 두 명 수준으로 제한되고 있었다. 하지만 2월 18일 31번째 확진자 발생 이후 대구 지역을 중심으로 상황은 급변하였다. 2020년 3월 26일 현재, 국내 9,241명의 누적 확진자 중 대구 지역 누적 확진자가 6,482명으로 전체 70.1%에 해당한다. 이 중, 신천지 관련 확진자는 4,391명으로 대구 지역 확진자의 67.7%에 해당한다. 대구 시민을 250만명, 대구 신천지 교인을 9,000명으로 가정하면, 대구 시민의 COVID-19 누적 감염율은 0.08%, 대구 신천지 교인의 누적 감염율은 48.78%로서, 대구 신천지 교인의 누적 감염율이 대구 시민의 누적 감염율 보다 약 583배 더 크다. 위 결과는 대구 지역 COVID-19 유행이 신천지 교인 집단이 고위험집단인 hotspot이 되어 대구 지역사회로 COVID-19가 전파되고 있음을 보여준다.

감염병 확산에 대한 수리적 연구 모델로 많이 이용되고 있는 구획 모델 (compartmental model) [3]은 일반적으로 전체 인구 집단을 감염 상태에 따라서 몇 가지의 상태로 나누는데, 대구 COVID-19 확산에서 나타나는 hotspot과 non-hotspot (신천지 교인이 아닌 지역사회)을 구현하기에는 적절하지 않다. 또한, 구획 모델에서 hotspot과 non-hotspot을 구현하기 위한 방법으로 두 개의 그룹으로 전체 인구 집단을 나누는 two-patch 모델 [4]이 제안되었지만 두 집단 간의 밀접 접촉을 통한 감염을 구현하는데 어려움이 존재한다.

우리는 위 문제들을 극복하기 위하여, Ferguson 등이 제안한 individual based model [5]을 이용하여 대구의 COVID-19 확산을 시뮬레이션 하였다. Individual based model에서 각 개인은 가구, 직장/학교, 공동체 (종교 및 친목 모임)에서 감염자와 접촉을 통하여 감염될 수 있으며, 이를 위하여 대구 인구와 동일한 크기의 가상 인구 집단을 생성하였다. Individual based model을 이용하여 지금까지의 대구 지역 COVID-19 유행을 이해하고, 향후 확산을 예측, 그리고 4월 6일로 예정된 개학이 COVID-19 확산에 어떤 영향을 미칠지 분석하는 것이 이 논문의 목표이다.

자료 및 방법 (materials and methods)

Individual based model [5]은 가구, 직장/학교, 공동체에서 사람간의 밀접 접촉을 통하여 감염병이 확산되는 과정을 시뮬레이션한다. 우리는 individual based model을 통하여 대구 지역 COVID-19 유행을 이해하고, 향후 확산을 예측하고자 하므로 대구 인구 규모의 가상 인구 집단을 생성하였다. 가상 인구 집단의 각 개인은 가구 정보, 직장/학교 정보, 공동체 정보를 갖는다. 우리는 마이크로데이터 통합서비스 [6]의 2015년 인구센서스 2% 샘플 자료로부터 2,171,000명의 가상 인구 집단을 생성하였다 (2015년 기준). 가구 정보 그리고 학생 및 직장 근무 여부는 인구센서스 자료에 기입된 각 개인의 연령, 학생 및 통근 여부를 이용하여 설정하였다. 직장 또는 학교에서의 밀접 접촉은 동일 사무실 또는 동일 교실 단위로 이루어진다고 가정하여, 학생의 교실 ID, 직장 근무자의 사무실 ID를 다음과 같이 가상으로 생성하였다: 교실 ID는 동일 연령, 동일 시군구의 학생들을 무작위로 선택하여 평균 30명이 같은 교실에 배정되도록 생성하였다. Fig. 1(a)는 대구 지역 각 교실의 학생 수에 따른 히스토그램을 보여준다. 사무실 ID는 시군구 구분 없이 대구 전체의 직장 근무자들을 무작위로 선택하여 평균 20명이 같은 사무실에 배정되도록 생성하였다. Fig. 1(b)는 대구 지역 각 사무실의 근무자 수에 따른 히스토그램을 보여준다.

Table 1은 위 방식으로 생성한 가상 인구 집단의 일부를 보여준다. 각 행은 한 명의 개인을 나타내고, 각 열은 감염병 확산 시뮬레이션에 이용되는 개인의 속성을 나타낸다. 가구 ID, 교실 ID, 사무실 ID가 같은 개인들은 각각 동일 가구, 동일 교실, 동일 사무실에 속한다. 교실 ID와 사무실 ID가 NA인 경우, 각각 학생이 아니고, 직장 근무자가 아닌 것을 의미한다. Hotspot 여부는 신천지 교인 여부를 나타내는 항목으로, 대구 전체 인구 중 무작위로 9,000명을 선택하여 hotspot에 할당하였다. Table 1의 마지막 열의 감염 상태는 해당 날짜의 각 개인의 감염 상태를 나타내는데, 가능한 감염 상태는 다음과 같다: COVID-19에 감염될 수 있는 감수성자-susceptible (S), 감염 후 잠복기 환자-latent (L), 감수성자를 감염시킬 수 있는 환자-infectious (I), 확진 후 격리 상태의 환자-hospitalization (H), 회복 혹은 사망-recovered (R). Fig. 2는 각 감염 상태 사이의 변화를 보여주는 다이어그램이다. $\lambda$는 감수성자의 감염 확률로서 다음과 같이 계산된다 [5].

$\lambda= \frac{\beta_{h}/3\cdot N_{h}^{I}}{\left( N_{h} \right)^{0.8}}+\frac{\beta_{w}/3\cdot N_{w}^{I}}{N_{w}}+\frac{\beta_{s}/3\cdot N_{s}^{I}}{N_{s}}+\frac{\beta_{hotspot}/3\cdot N_{hotspot}^{I}}{N_{hotspot}}$

$\beta_{h} (\beta_{w}, \beta_{s}, \beta_{hotspot})$은 가구 (직장, 학교, hotspot)에서 감염자를 만나고, 감염될 확률이다. Ferguson 등의 연구 결과 [5] 와 동일하게, 우리는 $\beta_{h}=\beta_{w}, \beta_{s}=2\beta_{h}$로 설정하였다. $N_{h} (N_{w}, N_{s}, N_{hotspot})$는 가구 (직장, 학교, hotspot)의 총 인원을 나타내며, $N_{h}^{I} (N_{w}^{I}, N_{s}^{I}, N_{hotspot}^{I})$는 해당 날짜의 가구 (직장, 학교, hotspot)의 총 감염자 수를 나타낸다. $1/\kappa=5.2$ [7], $1/\alpha=4.3$ [8], $1/\eta=14$는 각각 평균 잠복 기간(일), 증상 발현 후 확진까지의 평균 기간(일), 확진 후 회복까지의 평균 기간(일)을 의미한다.

우리는 2020년 2월 1일, hotspot에 10명의 감염자가 있었다고 초기 환자를 설정하였다. 31번 확진자는 2월 18일 확진 되었지만, 증상 시작일은 2월 7일로 알려졌으며, COVID-19의 잠복기를 고려하면 2월 1일 hotspot의 초기 환자들로부터 감염되었다고 설정하는 것은 합당한 가정이다. 우리는 일일 단위로 individual based model을 시뮬레이션 하였으며, 국내 다른 지역과 대구의 인구 이동은 고려하지 않았다. 즉 해외 및 국내 다른 지역으로부터 COVID-19 신규 감염자의 대구 유입은 없다고 가정되었다. 대구 지역 COVID-19 유행 이후 대중 교통 수단을 이용한 대구 지역으로의 인구 유입은 최소화되었기 때문에 위 가정은 합당하다고 판단된다.

결과 (results)

Individual based model, 그리고 $\beta_{hotspot}$과 $\beta_{h}$를 제외한 모든 파라미터 값에 대해서는 자료 및 방법 섹션에서 설명하였다. 우리는 2020년 3월 26일까지의 COVID-19 대구 지역 누적 확진자 (hotspot 4,391명, non-hotspot 2,091명)를 재현할 수 있도록, $\beta_{hotspot}$과 $\beta_{h}$를 설정하였다. 서로 다른 random seed를 이용하여 100번의 시뮬레이션을 하였으며, 이 결과의 중위값(median)이 누적 확진 결과를 재현하는지 확인하였다. $\beta_{hotspot}$과 $\beta_{h}$ 중, 대구 지역 전체 누적 확진자 재현에 더 높은 결정력을 갖는 파라미터는 $\beta_{hotspot}$이다. $\beta_{hotspot}$이 결정되면, hotspot의 누적 확진 결과가 재현되었고, 이후 $\beta_{h}$를 조정하면서 non-hotspot 누적 확진 결과를 재현하는 파라미터 값을 찾았다. 파라미터 결과는 $\beta_{hotspot}=3.06$, $\beta_{h}=0.33$이다. 파라미터 결과는 hotspot과 가구 및 직장에서의 감염 확률이 9배 이상 차이 나는 것을 보여준다. Fig. 3은 3월 26일까지의 누적 확진 결과와 individual based model 시뮬레이션 결과를 보여준다. 2월 말 신천지 교인에 대한 명단 확보 및 이들에 대한 대대적인 COVID-19 감염 검사 및 격리가 시작되었으므로, 위 시뮬레이션에서 우리는 2월 29일 이후로 $\beta_{hotspot}=0$으로 설정하였다. 또한, 신천지 교인에 대한 대대적인 감염 검사 이후로 증상 발현 후 확진까지의 평균 기간, $1/\alpha$가 짧아진 효과를 반영하기 위하여 2월 29일 이후 $1/\alpha$를 4.3이 아닌 2.7로 설정하였다.

3월 26일까지의 확진 결과를 재현하였는데, 이후 대구 지역의 COVID-19 확산을 예측하기 위하여, 우리는 다음 세 가지 시나리오를 고려하였다.

- 시나리오 I; 초/중/고 방학 유지
- 시나리오 II; 4월 6일 초/중/고 개학
- 시나리오 III; 4월 6일 초/중/고 개학 & 4월 6일 이후 증상 발현 후 확진까지의 평균 기간, $1/\alpha$는 4.3일로 다시 증가

위의 세 가지 시나리오에 대한 individual based model 시뮬레이션 결과는 Table 2와 Fig. 4-5에 정리하였다. 시나리오 I의 경우, 중위값 기준으로 대구 지역 최종 누적 확진자는 6,677명 (hotspot 4,394명, non-hotspot 2,283명)이고, 4월 26일 마지막 신규 확진자가 발생한다. 시나리오 II는 최종 누적 확진자 6,716명 (hotspot 4,394명, non-hotspot 2,322명)으로, 시나리오 I과 비교하여 (중위값 기준으로) non-hotspot, 즉 신천지 교인이 아닌 대구 시민 39명이 추가 감염된다. 마지막 신규 환자 발생일은 5월 3일로 시나리오 I과 비교하여 유행 종료가 7일 늦어진다. 시나리오 III은 누적 확진자 6,784명 (hotspot 4,394명, non-hotspot 2,390명)으로 시나리오 I과 비교하여 신천지 교인이 아닌 대구 시민 107명이 추가 감염된다. 마지막 신규 환자 발생일은 7월 27일로 유행 종료가 92일 늦어진다. Fig. 4는 각 시나리오 별 일일 누적 확진자를 나타낸다. 서로 다른 random seed를 이용한 100번의 시뮬레이션의 중위값과 90% 신뢰 구간(confidence interval)을 보여준다. Fig. 5는 hotspot과 non-hotspot의 누적 확진자에 대한 중위값 결과를 보여준다.

토의 (discussion)

이 논문을 통해 우리는 대구 지역의 COVID-19 확산이 보여준 특이성, 즉 신천지 교인(hotspot)과 비 신천지 교인(non-hotspot)의 누적 감염율이 약 583배라는 큰 차이를 나타내는 것을 설명할 수 있는 수리 모형으로 individual based model을 선택하였다. Individual based model은 가구, 직장/학교, 공동체 (종교 및 친목 모임)에서 사람간의 밀접 접촉을 통하여 감염병이 확산되는 과정을 시뮬레이션 하는데, 다른 감염병 확산 수리 모형과 비교하여 휴교 및 직장의 순환 근무 등 방제 정책의 효과를 적용하고 분석하기 용이한 장점이 있다.

우리는 individual based model을 이용하여 2020년 3월 26일까지의 대구 COVID-19 누적 확진 결과를 재현하였으며, hotspot과 non-hotspot이 서로 다른 감염 확률을 갖고 있는 것을 확인하였다 또한, 세 가지 시나리오에 대하여 3월 26일 이후 대구 지역의 COVID-19 확산을 예측하였다. 그 결과, 4월 6일로 예정된 초/중/고 개학을 하고 증상 발현 후 확진까지의 평균 기간이 4.3일로 다시 증가할 시나리오 III의 경우, 방학을 계속 유지하면서, 증상 발현 후 확진까지의 평균 기간이 2.7일인 시나리오 I의 경우와 비교하여 107명이 추가 감염되며, 대구 지역의 COVID-19 유행 종료는 92일 지연되는 것을 확인할 수 있었다.

위 결과는 해외 및 국내 다른 지역으로부터 COVID-19 신규 감염자가 대구로 유입되지 않는다고 가정한 결과이다. 향후 보다 정확한 예측 및 방제 정책 효과 분석을 위해서는 시뮬레이션을 전국으로 확대하고 지역간 인구 이동과 국외 감염 잠복기 환자의 입국을 고려해야 하는데, 현재 이에 대한 후속 연구를 준비하고 있다.

Acknowledgement

We thanks to all of the persons who were struggling in healthcare fields to overcome the COVID-19 outbreak. This study were performed under the research project named ‘Research and Development on Integrated Surveillance System for Early Warning of Infectious Diseases (RISEWIDs)’. The investigators of this project were Jaiyong Kim (Yonsei University), Sunworl Kim (National Medical Center), Eui Jung Kwon (National Health Insurance Review and Assessment Service), Dong Wook Kim (National Health Insurance Service), Moran Ki (National Cancer Center), Hyunjin Son (Pusan National University Hospital), Jong-Hun Kim (Sungkyunkwan University), Jin Yong Kim(Incheon Medical Center), Heeyoung Lee (Seoul National University Bundang Hospital), Boyoung Park (Hanyang University), Woo-Sik Son (National Institute for Mathematical Sciences), Jungsoon Choi (Hanyang University), Sunhwa Choi (National Cancer Center), Okyu Kwon (National Institute for Mathematical Sciences), Hyojung Lee (National Institute for Mathematical Sciences), Jong-Hoon Kim (International Vaccine Institute), Heecheon Kim (MISO INFO TECH), and Bo Youl Choi (Hanyang University). This project was supported by Government-wide R&D Fund Project for Infectious Disease Research (GFID), Republic of Korea (Grant No. HG18C0088).

References

1. World Health Organization. "Naming the coronavirus disease (COVID-19) and the virus that causes it." https://www. who. int/emergencies/diseases/novel-coronavirus-2019/technical-guidance/naming-the-coronavirus-disease-(covid-2019)-and-the-virus-that-causes-it (2020).
2. The New York Times. “China Confirms New Coronavirus Spreads From Humans to Humans.” <https://www.nytimes.com/2020/01/20/world/asia/coronavirus-china-symptoms.html> (2020).
3. Brauer, Fred. "Compartmental models in epidemiology." Mathematical epidemiology. Springer, Berlin, Heidelberg, 2008. 19-79.
4. Dowdy, David W., et al. "Heterogeneity in tuberculosis transmission and the role of geographic hotspots in propagating epidemics." Proceedings of the National Academy of Sciences 109.24 (2012): 9557-9562.
5. Ferguson, Neil M., et al. "Strategies for containing an emerging influenza pandemic in Southeast Asia." Nature 437.7056 (2005): 209-214.
6. Microdata Integrated Service. <https://mdis.kostat.go.kr/> (2020)
7. Li, Qun, et al. "Early transmission dynamics in Wuhan, China, of novel coronavirus–infected pneumonia." New England Journal of Medicine (2020).
8. Ki, Moran. "Epidemiologic characteristics of early cases with 2019 novel coronavirus (2019-nCoV) disease in Republic of Korea." Epidemiology and health (2020): e2020007.


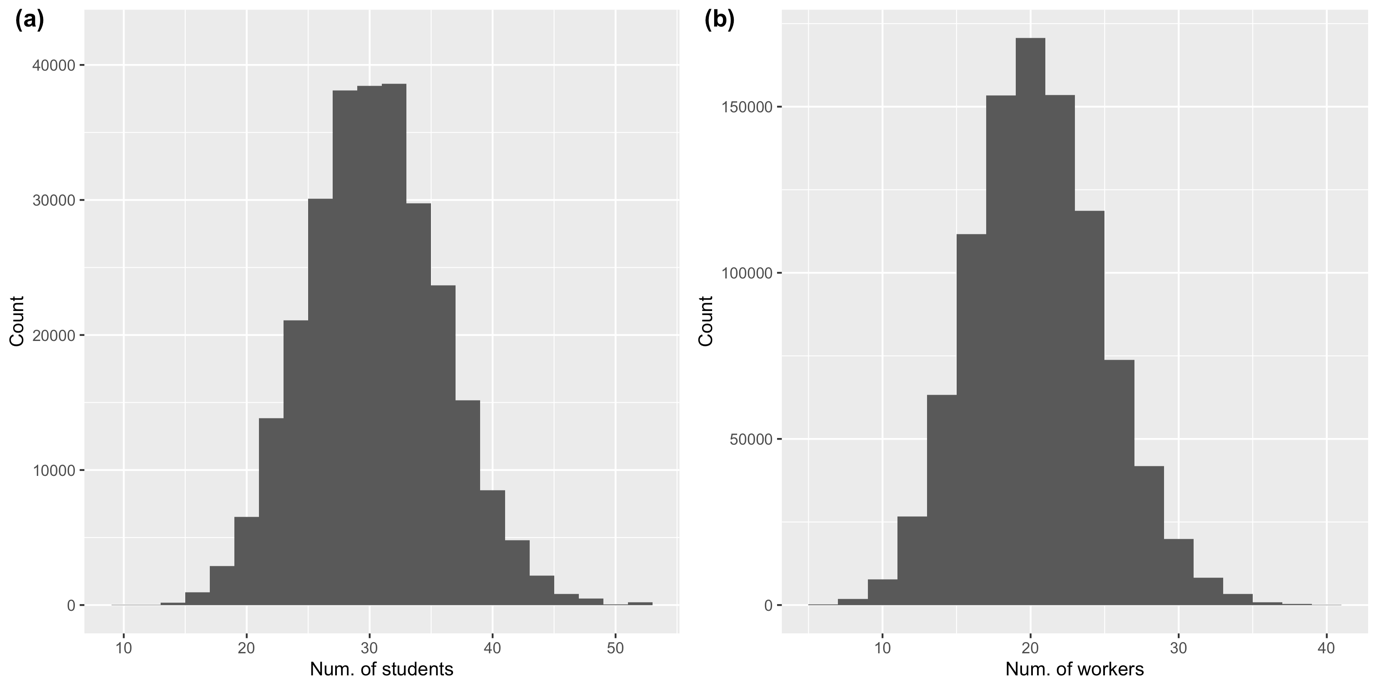


Fig. 1: (1) 대구 지역 교실의 학생 수에 따른 히스토그램, (b) 대구 지역 사무실의 근무자 수에 따른 히스토그램.

| 개인 ID | 가구 ID | 시군구 | 연령 | 교실 ID | 사무실 ID | hotspot | 감염 상태 |
| --- | --- | --- | --- | --- | --- | --- | --- |
| 1 | 1 | 대구 남구 | 48 | NA | 3 | FALSE | S |
| 2 | 1 | 남구 | 44 | NA | NA | TRUE | I |
| 3 | 1 | 남구 | 15 | 2 | NA | FALSE | S |
| 4 | 2 | 수성구 | 45 | NA | 3 | FALSE | S |
| 5 | 2 | 수성구 | 43 | NA | NA | FALSE | S |
| 6 | 2 | 수성구 | 17 | 25 | NA | FALSE | S |
| 7 | 3 | 수성구 | 51 | NA | 5 | FALSE | S |
| 8 | 3 | 수성구 | 50 | NA | NA | FALSE | S |
| 9 | 3 | 수성구 | 17 | 25 | NA | FALSE | S |

Table 1: 가상 인구 집단 테이블의 각 행은 한 명의 개인을 나타내고, 각 열은 감염병 확산 시뮬레이션에 이용되는 개인의 속성을 나타낸다. 가구 ID, 교실 ID, 사무실 ID가 같은 개인들은 각각 동일 가구, 동일 교실, 동일 사무실에 속한다. 교실 ID와 사무실 ID가 NA인 경우, 각각 학생이 아니고, 직장 근무자가 아닌 것을 의미한다. Hotspot 여부는 신천지 교인 여부를 나타내며, 감염 상태는 해당 날짜의 각 개인의 감염 상태를 표현한다.


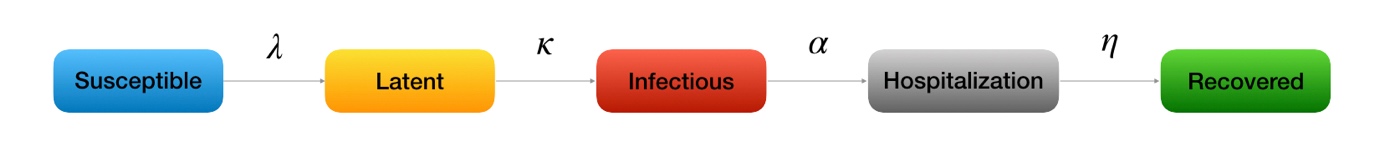


Fig. 2: 각 감염 상태 사이의 변화를 보여주는 다이어그램으로 가능한 감염 상태는 다음과 같다: COVID-19에 감염될 수 있는 감수성자-susceptible (S), 감염 후 잠복기 환자-latent (L), 감수성자를 감염시킬 수 있는 환자-infectious (I), 확진 후 격리 상태의 환자-hospitalization (H), 회복 혹은 사망-recovered (R).


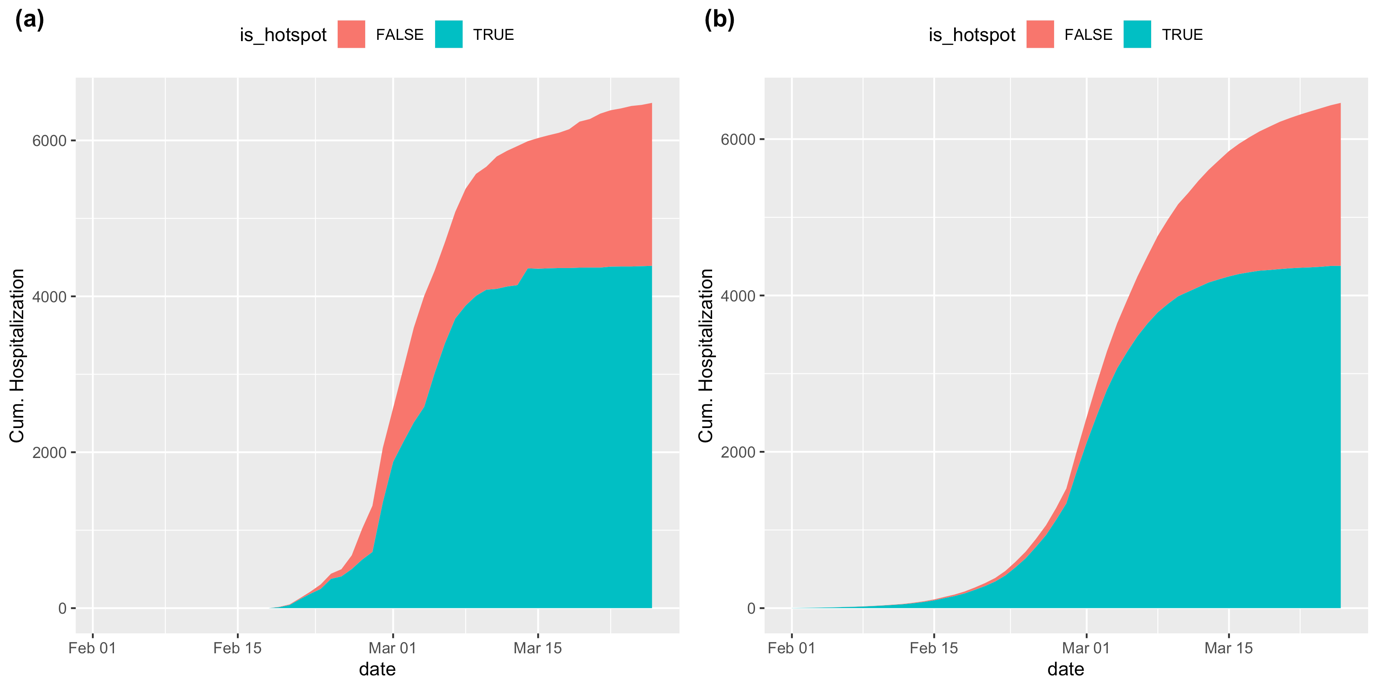


Fig. 3: (a) 대구 COVID-19 누적 확진자 수 (청록색은 hotspot, 빨간색은 non-hotspot), (b) individual based model 시뮬레이션 결과, 서로 다른 100개의 random seed에 대한 시뮬레이션 결과들의 중위값 (median).

|  | 시나리오 I | 시나리오 II | 시나리오 III |
| --- | --- | --- | --- |
| 설정 | 초/중/고 방학 유지 | 4월 6일 초/중/고 개학 | 4월 6일 초/중/고 개학,  4월 6일 이후 증상 발현 후 확진까지의 평균 기간은 4.3일로 다시 증가 |
| 최종 누적 확진자 수 | 6,677 (중위값 기준) | 6,716  (시나리오 I 대비 +39명) | 6,784  (시나리오 I 대비 + 107명) |
| 마지막 신규 확진자 발생일 | 4월 26일 (중위값 기준) | 5월 3일  (시나리오 I 대비 +7일) | 7월 27일  (시나리오 I 대비 +92일) |

Table 2: 각 시나리오 별 최종 누적 확진자 수 및 마지막 신규 확진자 발생일.


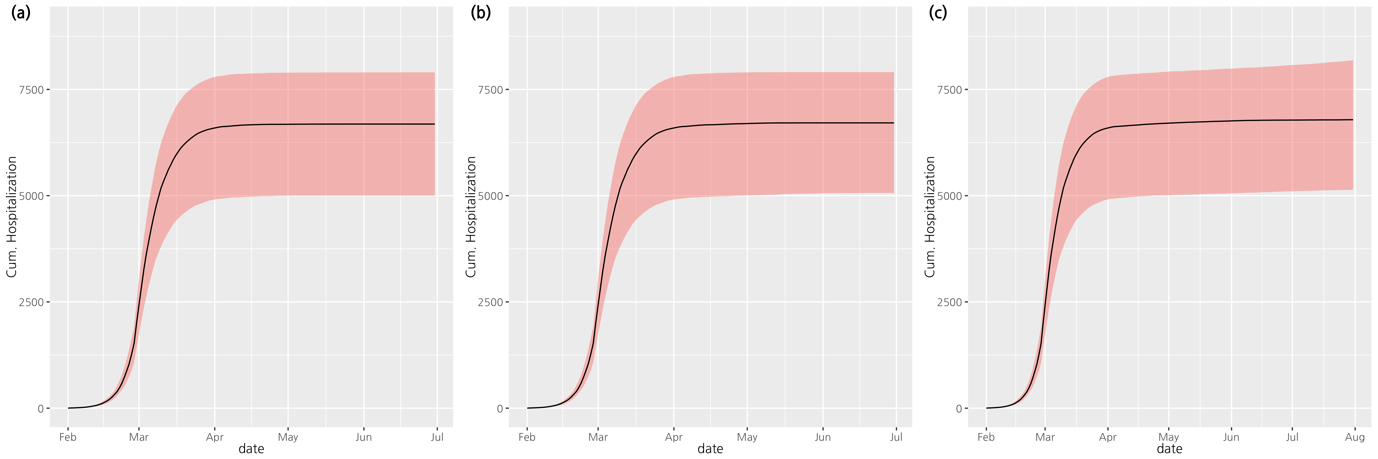


Fig. 4: 각 시나리오 별 일일 누적 확진자 (중위값과 90% 신뢰 구간) (a) 시나리오 I; 초/중/고 방학 유지, (b) 시나리오 II; 4월 6일 초/중/고 개학, (c) 시나리오 III; 4월 6일 초/중/고 개학, 4월 6일 이후 증상 발현 후 확진까지의 평균 기간이 4.3일로 다시 증가.


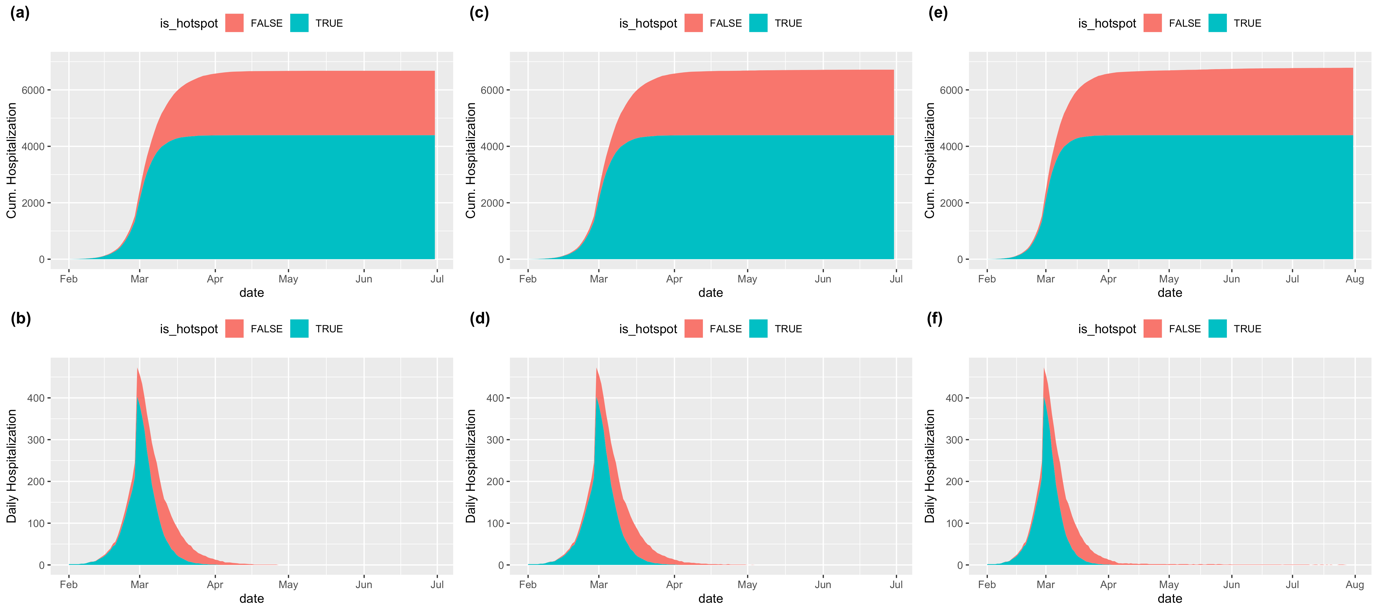


Fig. 5: (a) 시나리오 I (초/중/고 방학 유지)의 누적 확진자 수 (청록색은 hotspot, 빨간색은 non-hotspot, 서로 다른 100개의 random seed에 대한 시뮬레이션 결과들의 중위값), (b) 시나리오 I의 일일 신규 확진자 수, (c) 시나리오 II (4월 6일 초/중/고 개학)의 누적 확진자 수, (d) 시나리오 II의 일일 신규 확진자 수, (e) 시나리오 III (4월 6일 초/중/고 개학, 4월 6일 이후 증상 발현 후 확진까지의 평균 기간, 1/α는 4.3일로 다시 증가)의 누적 확진자 수, (f) 시나리오 III의 일일 신규 확진자 수.
